# Effect of Microvascular Invasion on the Prognosis in Hepatocellular Carcinoma and Analysis of Related Risk Factors: A Two-Center Study

**DOI:** 10.3389/fsurg.2021.733343

**Published:** 2021-11-17

**Authors:** Wang Yanhan, Lu Lianfang, Liu Hao, Ding Yunfeng, Song Nannan, Lin Fanfan, Zhu Chengzhan, Wu Meilong, Sun Chuandong

**Affiliations:** ^1^Department of Hepatobiliary and Pancreatic Surgery, The Affiliated Hospital of Qingdao University, Qingdao, China; ^2^Department of Operation Room, The Affiliated Hospital of Qingdao University, Qingdao, China; ^3^Shandong Key Laboratory of Digital Medicine and Computer Assisted Surgery, The Affiliated Hospital of Qingdao University, Qingdao, China; ^4^School of Clinical Medicine, Tsinghua University, Beijing, China

**Keywords:** hepatocellular carcinoma, microvascular invasion, prognosis, risk factors, recurrence

## Abstract

**Objective:** Microvascular invasion is considered to initiate intrahepatic metastasis and postoperative recurrence of hepatocellular carcinoma (HCC). We aimed to analyze the effect of MVI on the prognosis in HCC and identify related risk factors for microvascular invasion (MVI).

**Methods:** The clinical data of 553 HCC patients who underwent liver surgery at Qingdao University from January 2014 to December 2018 and 89 patients at Beijing Tsinghua Changgung Hospital treated between October 2014 and October 2019 were collected retrospectively. We explored the impact of MVI on the prognosis of patients with HCC using Kaplan-Meier analysis. We conducted logistic regression analysis to identify variables significantly related to MVI.

**Results:** Pathological examination confirmed the presence of MVI in 265 patients (41.3%). Six factors independently correlated with MVI were incorporated into the multivariate logistic regression analysis: Edmondson-Steiner grade [odds ratio (OR) = 3.244, 95%CI: 2.243–4.692; *p* < 0.001], liver capsule invasion (OR = 1.755; 95%CI: 1.215–2.535; *p* = 0.003), bile duct tumor thrombi (OR = 20.926; 95%CI: 2.552–171.553; *p* = 0.005), α-fetoprotein (> 400 vs. < 400 ng/ml; OR = 1.530; 95%CI: 1.017–2.303; *p* = 0.041), tumor size (OR = 1.095; 95%CI: 1.027–1.166; *p* = 0.005), and neutrophil-lymphocyte ratio (OR = 1.086; 95%CI: 1.016–1.162; *p* = 0.015). The area under the receiver operating characteristic curve (AUC) was 0.743 (95%CI: 0.704–0.781; *p* < 0.001), indicating that our logistic regression model had significant clinical usefulness.

**Conclusions:** We analyzed the effect of MVI on the prognosis in HCC and evaluated the risk factors for MVI, which could be helpful in making decisions regarding patients with a high risk of recurrence.

## Introduction

Primary liver cancer is the third leading cause of cancer-related deaths worldwide with more than 900,000 new cases annually (1). Hepatocellular carcinoma (HCC) is the major pathologic category of primary liver cancer (PLC) and accounts for ~90% of all pathological types. Although treatments for HCC have become increasingly diversified with the development of targeted therapy and immunotherapy (2), the prognosis remains poor (3). Hepatectomy and liver transplantation are the preferred therapies for HCC, according to the international guidelines. However, <30% of the patients receive surgical treatment. The study of Llovet et al. reported that the 5-year recurrence and metastasis rates were 40–70% after surgery (4). The 5-year recurrence rate after surgery is as high as 80% for tumors larger than 5 cm and 40–50% for tumors smaller than 5 cm (5). Early recurrence is a key factor affecting the overall survival (OS) in patients with HCC. Therefore, screening for early metastasis and predictors of recurrence is important.

Early recurrence is closely related to aggressive tumor cell growth. Microvascular invasion (MVI) is defined as the presence of tumor cells in the portal veins, large capsule vessels, or vascular spaces lined by endothelial cells (6), which can only be confirmed by postoperative histological examination. MVI can lead to the spread of tumor cells within the liver or distant metastasis. MVI is reportedly a negative factor that results in decreased long-term survival and early recurrence (7). The occurrence rate of MVI in patients with HCC ranges from 15 to 57% (8). For patients with HCC smaller than 5 cm, the disease-free survival (DFS) and OS rates are worse in patients with a high risk of MVI than in those with a low risk after radiofrequency ablation treatment (9). Different surgical resection methods and surgical margins also affect the prognosis of patients with HCC and MVI. Patients with surgical margins ≥1 cm have a lower postoperative recurrence rate and better long-term prognosis than those with a surgical margin <1 cm (10). According to a study by Ueno et al., anatomical segmentation is currently recommended as the standard surgical procedure for patients with HCC and MVI (11). However, the pathological detection rate of MVI in HCC and the impact on patient survival was depending on the tissue sampling protocol (12). The seven-point sampling protocol may not satisfy the detection of all the existing MVI. Therefore, precise identification of MVI is beneficial in choosing more suitable therapeutic decisions.

In our study, the clinicopathological data of patients with HCC with MVI were retrospectively analyzed. We explored the effect of MVI on the prognosis and identified the risk factors associated with MVI to provide clues identifying the HCC with a high risk of recurrence.

## Materials and Methods

### Patients

We retrospectively collected the clinicopathological data of 553 patients from the Affiliated Hospital of Qingdao University between January 2014 and December 2018 and 89 patients from the Beijing Tsinghua Changgung Hospital who underwent primary hepatectomy for HCC between October 2014 and October 2019.

This study was approved by the Ethics Committee of the Affiliated Hospital of Qingdao University and the Ethics Committee of the Beijing Tsinghua Changgung Hospital.

We chose the Guidelines for Diagnosis and Treatment of Primary Liver Cancer in China (2020 Edition) (13) as the basic diagnosis of HCC. The major inclusion criteria were as follows: (1) liver cancer without distant metastasis on preoperative imaging examination, (2) hepatectomy with tumor confirmed as HCC by histology, (3) available pathological sections and pathological report data, and (4) survival and recurrence information obtained by follow-up.

The exclusion criteria were as follows: (1) preoperative therapy before liver section, (2) incomplete removal of the tumor, (3) unclear pathological type of PLC, (4) incomplete laboratory data and follow-up information, and (5) history of other malignant tumors.

The follow-up time was until October 1, 2020. All patients were followed up for more than 1 year.

### Statistical Analysis

All statistical analyses were performed using IBM SPSS Statistics version 22 (SPSS Inc., Chicago, IL, USA). SPSS Statistics version 22 was used for the survival analysis, univariate analysis, and multivariate logistic regression analysis. Survival analysis was performed using the Kaplan-Meier method and log-rank test. The coefficient of skewness, coefficient of kurtosis, and histogram were combined to determine whether the continuous variables fit the normal distribution. If so, we used the *M* ± *SD* to represent continuous variables. An independent sample t-test was used to compare the two groups. Means and quaternary intervals (mediums, interquartile ranges) were used to describe the continuous variables that did not conform to a normal distribution, and the nonparametric Mann-Whitney U rank-sum test was used for comparisons between the two groups. Categorical variables were displayed as the rate or constituent ratio, which were compared using the chi-square test or Fisher's exact test. Univariate analysis was used to assess the significance of each variable. All variables with statistical significance in the univariate logistic regression analysis were incorporated into a multivariate logistic regression analysis. Odds ratios (ORs) and 95% CIs were calculated. Statistical significance was set at *p* < 0.05.

## Results

### Clinical and Demographic Characteristics

According to the inclusion and exclusion criteria, 642 HCC patients were included in our study. All patients underwent preoperative imaging examinations. After a detailed clinical evaluation, liver resection was performed. All resected surgical specimens were pathologically assessed for subsequent analyses. The presence of MVI was confirmed in 265 patients (41.3%).

### MVI Affected the Prognosis in HCC

**Figure 1:**
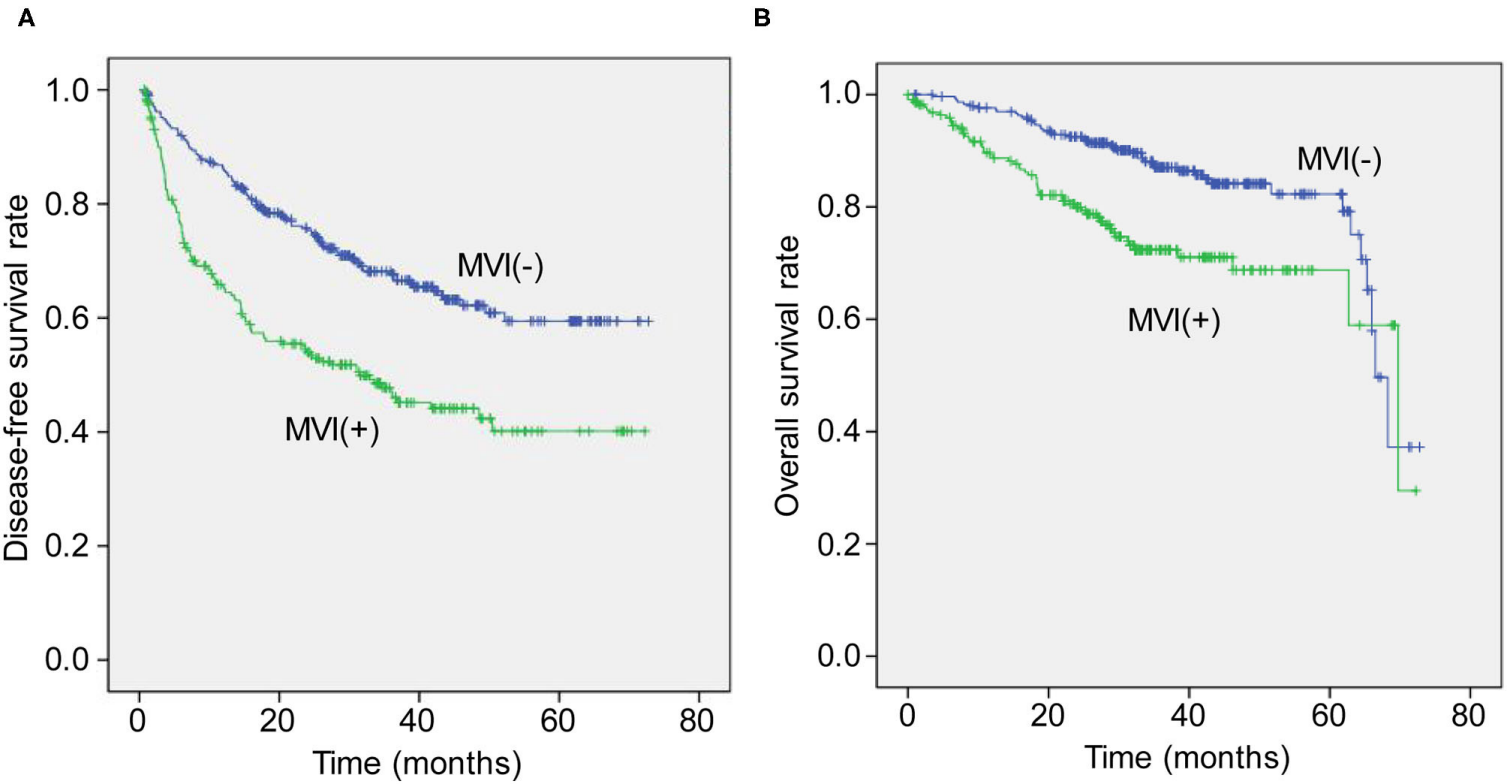
The Kaplan-Meier curve for disease-free survival **(A)** and overall survival **(B)** time between groups with and without microvascular invasion after resection.

In the MVI positive (MVI+) group, there were 137 cases of recurrence, while 128 cases had no recurrence. In the MVI negative (MVI–) group, there were 134 cases of recurrence while 243 cases had no recurrence. The DFS rates at 1, 3, and 5 years in the MVI+ group were 97.3, 89.9, and 80.2%, respectively, while they were 98.1, 91.4, and 85.5% in the MVI– group, respectively (*p* < 0.05, Figure 1A).

Seventy-one patients died in the MVI+ group, while 65 patients died in the MVI– group. The overall survival rates at 1, 3, and 5 years in the MVI+ group were 98.1, 93.3, and 87.8%, respectively, while in the MVI– group, they were 98.9, 94.8, and 92.2%, respectively (*p* < 0.05, Figure 1B).

### Related Risk Factors for MVI

**Figure 2:**
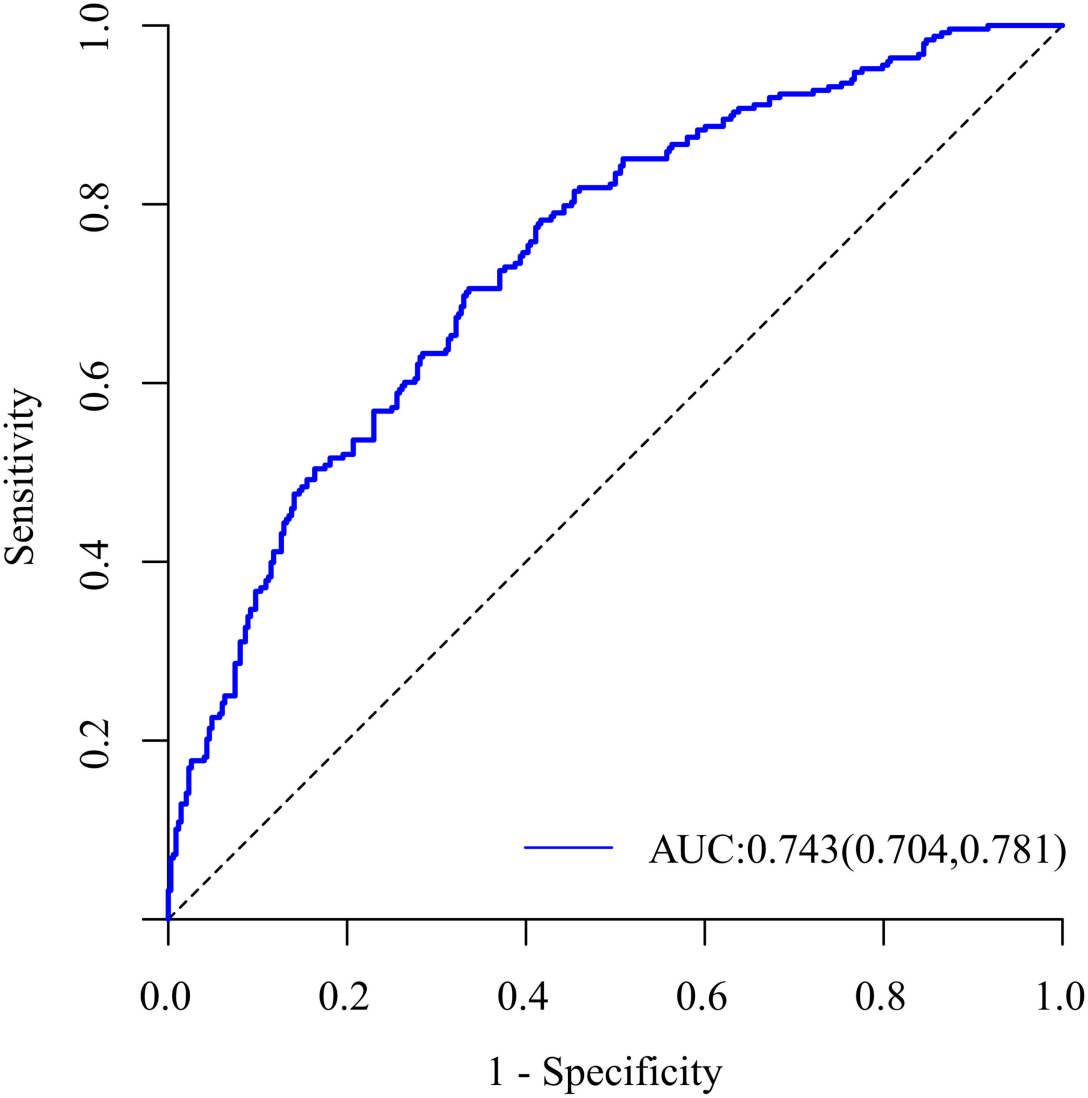
The receiver operating characteristic curve of the logistic regression model.

Univariate analysis revealed that tumor size, neutrophil count, neutrophil-lymphocyte ratio (NLR), platelet-lymphocyte ratio (PLR), γ-glutamyltransferase (GGT), Edmondson-Steine grade, liver capsule invasion, satellite nodules, bile duct tumor thrombi (BDTT), and α-fetoprotein (AFP, >400 vs. <400 ng/ml) were associated with MVI (Tables 1, 2). Potentially related factors were then enrolled in a multivariable logistic regression analysis selected by the stepwise forward method. The Edmondson-Steiner grade (OR = 3.244, 95%CI: 2.243–4.692, *p* < 0.001), liver capsule invasion (OR = 1.755, 95%CI: 1.215–2.535, *p* = 0.003), BDTT (OR = 20.926, 95%CI: 2.552–171.553, *p* = 0.005), AFP (OR = 1.53; 95%CI: 1.017–2.303; *p* = 0.041), tumor size (OR = 1.095; 95%CI: 1.027–1.166; *p* = 0.005), and NLR (OR = 1.086; 95%CI: 1.016–1.162; *p* = 0.015) were independent risk factors for MVI (Table 3). The area under the receiver operating characteristic (ROC) curve (AUC) was 0.743 (95%CI: 0.704–0.781; *p* < 0.001, Figure 2), showing that the predictive effect of the model was good.

**Table 1:** Univariate analysis of continuous variables.

**Variable**	**Total** **(***n*** = 642)**	**MVI–** **(***n*** = 377)**	**MVI+** **(***n*** = 265)**	***p***
Age (y)	57.69 ± 10.18	57.6 ± 9.91	57.82 ± 10.57	0.787
Tumor size (cm)	3.5 (2.5–6)	3.2 (2.3–5)	4.5 (3–7)	<0.001
Neutrophils (10^9^/L)	2.96 (2.39–3.88)	2.8 (2.31–3.67)	3.13 (2.51–4.26)	<0.001
Lymphocytes (10^9^/L)	1.6 (1.21–2.08)	1.63 (1.25–2.13)	1.56 (1.12–2.03)	0.055
PLT (10^9^/L)	158.5 (120–202)	157 (121–198)	162 (120–205)	0.551
INR	0.97 (0.91–1.04)	0.97 (0.91–1.03)	0.97 (0.9–1.04)	0.856
PT (s)	10.8 (10.1–11.6)	10.8 (10.2–11.5)	10.8 (10–11.6)	0.703
Alb (g/L)	40.85 ± 4.48	40.78 ± 4.4	40.95 ± 4.61	0.622
Tbil (umol/L)	17.54 (12.76–22.8)	17.21 (12.25–22.49)	18.25 (13.23–23)	0.109
ALT (U/L)	33 (23–51)	34.5 (23.05–53.5)	32 (22–51)	0.407
AST (U/L)	30 (22.1–43)	29.85 (22–40)	31 (22.8–47)	0.075
GGT (U/L)	38 (25–70)	36 (24–63)	45 (26–79)	0.006
NLR	1.86 (1.34–2.62)	1.74 (1.26–2.4)	2.06 (1.53–2.87)	<0.001
PLR	99.38 (73.6–131.4)	97.53 (71.13–126.42)	104.86 (76.33–144.6)	0.011

*MVI, microvascular invasion; PLT, platelets; INR, international normalized ratio; PT, prothrombin; Alb, albumin; Tbil, total bilirubin; ALT, alanine aminotransferase; AST, aspartate aminotransferase; GGT, γ-glutamyltransferase; NLR, neutrophil-lymphocyte ratio; PLR, platelet-lymphocyte ratio*.

**Table 2:** Univariate analysis of categorical variables.

**Variable**		**Total**	**MVI–**	**MVI+**	***p***
		**(*n* = 642)**	**(*n* = 377)**	**(*n* = 265)**	
Sex	Female	522 (81.31)	302 (80.11)	220 (83.02)	0.351
	Male	120 (18.69)	75 (19.89)	45 (16.98)	
Edmondson-Steiner grade	I/II	421 (65.68)	293 (77.72)	128 (48.48)	<0.001
	III/IV	220 (34.32)	84 (22.28)	136 (51.52)	
Liver capsule invasion	Absent	323 (50.47)	223 (59.47)	100 (37.74)	<0.001
	Present	317 (49.53)	152 (40.53)	165 (62.26)	
Number of tumors	Single	569 (88.77)	337 (89.63)	232 (87.55)	0.494
	≥2	72 (11.23)	39 (10.37)	33 (12.45)	
Satellite nodules	Absent	560 (87.50)	344 (91.49)	216 (81.82)	<0.001
	Present	80 (12.50)	32 (8.51)	48 (18.18)	
Cirrhosis	Absent	216 (33.70)	126 (33.51)	90 (33.96)	0.905
	Present	425 (66.30)	250 (66.49)	175 (66.04)	
BDTT	Absent	623 (97.50)	375 (99.73)	248 (94.30)	<0.001
	Present	16 (2.50)	1 (0.27)	15 (5.70)	
Surgical options	Open	419 (65.47)	249 (66.05)	170 (64.64)	0.712
	Laparoscope	221 (34.53)	128 (33.95)	93 (35.36)	
AFP, ng/ml	≤400	469 (73.28)	297 (79.20)	172 (64.91)	<0.001
	>400	171 (26.72)	78 (20.80)	93 (35.09)	
HBsAg	Negative	109 (17.06)	59 (15.73)	50 (18.94)	0.289
	Positive	530 (82.94)	316 (84.27)	214 (81.06)	

*MVI, microvascular invasion; BDTT, bile duct tumor thrombus; AFP, alpha-fetoprotein; HBsAg, type B hepatitis surface antigen*.

**Table 3:** Multivariate regression analysis of data for the presence of microvascular invasion.

**Variable**	**β**	**OR (95%CI)**	***p***
Edmondson-Steiner grade	1.177	3.244 (2.243–4.692)	<0.001
Liver capsule invasion	0.563	1.755 (1.215–2.535)	0.003
BDTT	3.041	20.926 (2.552–171.553)	0.005
AFP (>400 vs. ≤400 ng/ml)	0.426	1.530 (1.017–2.30)	0.041
Tumor size, cm	0.090	1.095 (1.027–1.166)	0.005
NLR	0.083	1.086 (1.016–1.162)	0.015

*BDTT, bile duct tumor thrombus; AFP, alpha-fetoprotein; NLR, neutrophil-lymphocyte ratio; OR, odds ratio; CI, confidence interval*.

## Discussion

In HCC, MVI is a key risk factor for early recurrence after surgical resection (14). Our study found that the presence of MVI in HCC suggested a poor prognosis. Both DFS and OS times in MVI+ patients were worse than those in MVI– patients. The presence of MVI often influences the formulation of clinical treatment plans and postoperative efficacy. Surgical resection is the first choice for patients with HCC. Ideally, hepatectomy requires complete removal of the tumor while preserving adequate liver function. Using the Couinaud segmentation method, the surgeon can completely remove the tumors, the liver segment, and the micrometastases through the portal system. Previous studies have indicated that anatomical liver resection is the preferred option for patients with a high suspicion of MVI (15, 16). If patients have a low risk of MVI, local resection may be selected to reduce the risk of bleeding and infection, improving the short-term prognosis (17). Therefore, preoperative prediction, intraoperative management, and postoperative intervention for MVI have become research hotspots. However, the MVI detection rate relied on tissue sampling protocol which may fail to obtain the existing MVI (12). This study aimed to identify independent risk factors related to the occurrence of MVI, in order to provide clues identifying the patients with a high risk of recurrence. Our statistical analysis of 642 HCC patients revealed six independent predictive factors for MVI in HCC, particularly Edmondson-Steiner grade, capsule invasion, BDTT, AFP, tumor size, and NLR.

The occurrence of MVI is closely related to the tumor size (18). Pawlik et al. (9) found that tumor diameter was positively correlated with the incidence of MVI. This is consistent with the results of our study. Shi et al. (19) analyzed the pathological specimens of 113 cases of single HCC and found that the incidence of MVI significantly increased when the tumor diameter was >3 cm. In general, a larger tumor size often indicates a higher risk of MVI (20, 21). To prevent excessive loss of information, tumor size was treated as a continuous variable in our study. Regarding the relationship between tumor differentiation and MVI, some scholars believe that tumor differentiation is negatively correlated with the incidence of MVI (22). Our study concluded that Edmondson-Steiner grade and liver capsule invasion were independent risk factors for MVI in HCC. Tumor differentiation can only be determined by postoperative pathology, while tumor size and liver capsule invasion can be preliminarily assessed by imaging examination before surgery. Therefore, tumor size and liver capsule invasion may have a higher practical value in the preoperative diagnosis of MVI than the degree of tumor differentiation.

Currently, serum AFP level testing is one of the most common clinical laboratory procedures for the diagnosis of HCC. We chose AFP levels above 400 ng/ml as an important predictor of MVI, as revealed by previous research (23), and found that the MVI+ group had higher AFP levels than the MVI– group. Some studies have also shown that an AFP level over 100 ng/ml is an important indicator for predicting MVI (24). AFP levels are now widely regarded as a predictor of MVI, while the threshold of the AFP level used to predict MVI is still controversial.

According to previous reports, inflammation plays an important role in the occurrence, development, invasion, and metastasis of tumors (25). Many inflammatory markers in laboratory tests have been confirmed to have predictive value for the development of MVI (18, 25). The NLR was independently related to MVI, and this has been confirmed in a number of nomograms that calculated the predictive probability of preoperative MVI in HCC patients (26, 27). In our study, there was a significant difference in NLR levels between HCC patients with and without MVI. This is consistent with the results of previous studies. However, most of the inflammatory cell indicators in our cases were within the normal range, and these kinds of variables with a small range of numerical fluctuations depend on a large sample size to obtain more accurate results. The relationship between inflammatory response and the occurrence of MVI needs to be further investigated.

HCC with BDTT is rarely seen clinically, and the incidence of BDTT accounts for 1.2–9% of HCC (28). HCC with BDTT has aggressive characteristics, and its long-term prognosis is extremely dismal (29). A study reported that HCC patients with BDTT tended to have a higher probability of having MVI (30). We also found that BDTT is an important factor in predicting MVI. Due to the small sample size of patients with BDTT in this study, its value in predicting MVI needs further investigation.

In contrast with other studies, our study included patients from two centers and was constructed based on routine laboratory parameters that are more accessible and easily interpreted. However, our study has limitations as a retrospective study. First, although some individual factors such as Edmondson-Steiner grade were statistically significant, they can only be confirmed through postoperative pathology. This greatly limits the guidance significance of preoperative prediction models for treatment. Second, ~80% of patients were complicated with hepatitis virus infection in our study. Whether this model can be extended to other patients with HCC in Western countries is still unknown. It is still necessary to carry out multicenter, large-sample, prospective studies to achieve an accurate diagnosis of MVI and further explore its clinical significance.

In conclusion, we found that MVI influenced the prognosis in HCC and we analyzed independent risk factors for the occurrence of MVI. We hope that the results of this study will provide clues identifying the patients with a high risk of recurrence other than MVI alone.

## Data Availability Statement

The original contributions presented in the study are included in the article/Supplementary Material, further inquiries can be directed to the corresponding authors.

## Ethics Statement

The studies involving human participants were reviewed and approved by the Ethics Committee of the Affiliated Hospital of Qingdao University and the Ethics Committee of the Beijing Tsinghua Changgung Hospital. Written informed consent for participation was not required for this study in accordance with the national legislation and the institutional requirements.

## Author Contributions

WY and ZC designed the study, analyzed the results, and wrote the manuscript. SC and WM provided strategic guidance. LL, LH, DY, SN, and LF collected the data. All authors commented on the manuscript, contributed to the article, and approved the submitted version.

## Funding

This work was supported by the Shandong Higher Education Young Science and Technology Support Program (Grant Number 2020KJL005).

## Conflict of Interest

The authors declare that the research was conducted in the absence of any commercial or financial relationships that could be construed as a potential conflict of interest.

## Publisher's Note

All claims expressed in this article are solely those of the authors and do not necessarily represent those of their affiliated organizations, or those of the publisher, the editors and the reviewers. Any product that may be evaluated in this article, or claim that may be made by its manufacturer, is not guaranteed or endorsed by the publisher.
